# The diagnostic value of quantitative analysis of ASL, DSC-MRI and DKI in the grading of cerebral gliomas: a meta-analysis

**DOI:** 10.1186/s13014-020-01643-y

**Published:** 2020-08-24

**Authors:** Jixin Luan, Mingzhen Wu, Xiaohui Wang, Lishan Qiao, Guifang Guo, Chuanchen Zhang

**Affiliations:** 1 Department of Radiology, Liaocheng People’s Hospital, Shandong First Medical University & Shandong Academy of Medical Sciences, 67, Dongchang West Road, Liaocheng District, 252000 Shandong Province China; 2 Department of Science and Education, Liaocheng People’s Hospital, Shandong First Medical University & Shandong Academy of Medical Sciences, 67, Dongchang West Road, Liaocheng District, 252000 Shandong Province China; 3 School of Mathematics, Liaocheng University, Liaocheng District, 252000 Shandong Province China

**Keywords:** Gliomas, Grading, MRI, Meta-analysis

## Abstract

**Objective:**

To perform quantitative analysis on the efficacy of using relative cerebral blood flow (rCBF) in arterial spin labeling (ASL), relative cerebral blood volume (rCBV) in dynamic magnetic sensitivity contrast-enhanced magnetic resonance imaging (DSC-MRI), and mean kurtosis (MK) in diffusion kurtosis imaging (DKI) to grade cerebral gliomas.

**Methods:**

Literature regarding ASL, DSC-MRI, or DKI in cerebral gliomas grading in both English and Chinese were searched from PubMed, Embase, Web of Science, CBM, China National Knowledge Infrastructure (CNKI), and Wanfang Database as of 2019. A meta-analysis was performed to evaluate the efficacy of ASL, DSC-MRI, and DKI in the grading of cerebral gliomas.

**Result:**

A total of 54 articles (11 in Chinese and 43 in English) were included. Three quantitative parameters in the grading of cerebral gliomas, rCBF in ASL, rCBV in DSC-MRI, and MK in DKI had the pooled sensitivity of 0.88 [95% CI (0.83,0.92)], 0.92 [95% CI (0.83,0.96)], 0.88 [95% CI (0.82,0.92)], and the pooled specificity of 0.91 [95% CI (0.84,0.94)], 0.81 [95% CI (0.73,0.88)], 0.86 [95% CI (0.78,0.91)] respectively. The pooled area under the curve (AUC) were 0.95 [95% CI (0.93,0.97)], 0.91 [95% CI (0.89,0.94)], 0.93 [95% CI (0.91,0.95)] respectively.

**Conclusion:**

Quantitative parameters rCBF, rCBV and MK have high diagnostic accuracy for preoperative grading of cerebral gliomas.

Gliomas are the most common primary tumors of the central nervous system, accounting for about 45% of all intracranial tumors [1]. On pathology, Gliomas can be divided into low-grade gliomas (LGGs, including WHO I ~ II) and high-grade gliomas (HGGs, including WHO III ~ IV) according to their histological and molecular features [2]. Surgical resection combined with radiotherapy and chemotherapy are still the basic treatment for gliomas. LGGs grow slowly and has a favorable prognosis in general [3]; but HGGs are more aggressive with a 5-year relative survival rate of 15–58% for anaplastic astrocytomas and of 6–22% for glioblastomas depending upon their age at diagnosis and various other prognostic factors [4]. Therefore, accurate assessment of the pathological grade of gliomas before surgery is of great clinical significance to determine the surgical resection range and to improve the survival rate of patients.

In the course of gliomas progression, the microstructures of tumors (tumor cell density, cell proliferation activity, and microvessel density, etc.) will undergo tremendous changes, reflecting the changes in histopathological characteristics of tumors [5]. Traditional morphological magnetic resonance imaging (MRI) can estimate the extent of histopathological differentiation of tumors based on cytotoxic edema, hemorrhage, necrosis, heterogeneity of signal intensity, degree and range of signal enhancement. However, studies have shown that the enhancement of gliomas is not completely consistent with tumor grade [6]. Roy et al. [7] reported that the sensitivity of conventional MRI to differentiate high-grade gliomas from low-grade gliomas ranged from 55.1 to 83.3%.

With the continuous development of MRI technology, multi-modal MRI technology has been used to evaluate the biological characteristics of gliomas from different perspectives and has potential application value in the grading of gliomas. Among them, artery spin labeling (ASL) and dynamic magnetic sensitivity enhanced perfusion imaging (DSC-MRI) are perfusion-weighted imaging techniques, while diffusion kurtosis imaging (DKI) is a diffusion magnetic resonance imaging technique. Quantitative perfusion parameters such as relative cerebral blood flow (rCBF) in ASL, relative cerebral blood volume (rCBV) in DSC-MRI and mean kurtosis (MK) in DKI are receiving more attention in the clinical application of preoperative grading of gliomas. Compared with low-grade gliomas, high-grade gliomas have a more abundant blood supply, so hemodynamic perfusion parameters will increase significantly [8]. The cellular pleomorphism and nuclear polymorphism in high-grade gliomas are more marked than those in low-grade gliomas, and the parameters associated with water molecular diffusion are also larger [9].

Most previous researches only utilized perfusion imaging or diffusion imaging to investigate the grading of gliomas, focusing on meta-analysis of diagnostic accuracy. The meta-analysis of quantitative parameters of the above imaging methods are lacking. Moreover, previous efforts only focused on the meta-analysis of diagnostic accuracy and lacked the meta-analysis on quantitative parameters. Due to the small sample size and incomplete parameters of individual studies, the reliability and repeatability of the technology are still unclear. Therefore, we propose a large sample-size comprehensive meta-analysis to resolve the conflicting findings in different studies and to evaluate the diagnostic performance of the quantitative perfusion and diffusion parameters in gliomas grading.

## Materials and methods

### Literature retrieval

A thorough search for literature from 2005 to 2019 relating to ASL, DSC-MRI or DKI in the grading of cerebral gliomas was performed, using sources from PubMed, Embase, Web of Science, CBM, China National Knowledge Infrastructure (CNKI), Wanfang Database. English search keywords were (astrocytoma or glioblastoma or glioma tumor or astrocytic tumor or gliomas or oligodendroglioma or oligodendroglial tumor) and (DKI or Diffusional Kurtosis or Kurtosis Imaging or kurtosis or DSC-MRI or Dynamic susceptibility contrast-enhanced MRI or Dynamic Susceptibility Contrast or DSC or rCBV or rCBF or ASL or arterial spin-labeling or perfusion or Continuous ASL perfusion or PASL or 3DpCASL or three-dimensional pseudo-continuous arterial spin labeling). In order to avoid missing documents, the combination of electronic search and manual search were performed.

### Literature inclusion and exclusion criteria

#### Inclusion criteria

(1) ASL, DSC-MRI or DKI were used to differentiate gliomas of different grades; (2) At least one quantitative parameter of rCBF, rCBV and MK could be extracted or calculated from the study; (3) Only pathological diagnoses were included; (4) All subtypes of gliomas were included; (5) Fourfold table values of diagnostic tests can be obtained directly or indirectly, i.e. true-positive, true-negative, false-positive, and false-negative; (6) The quality evaluation scores of the included studies were at least 9 since high-quality studies are the basis for reliable meta-analysis.

#### Exclusion criteria

(1) animal experiments, such as animal experiments of rats; (2) any unpublished conference abstracts, comments, duplication of literature or research; (3) similar studies written by the same author; (4) lack of key data; (5) use of other imaging methods (such as CT, PET, etc.).

### Data extraction from literature

The basic information includes first author’s name, country, the time of publication, patient age, tumor grade, number of cases, instrument type and field strength, journal of publication, methods, sequence and so on. Diagnostic information includes sensitivity, specificity, Fourfold table and the ROC curve with the corresponding area under the curve (AUC) value. If the information could not be obtained directly, the statistics were performed with the number of HGG and LGG cases and the sensitivity and specificity provided by the literature using RevMan 5.3 Software [10]. For articles providing sample size, median, extremum or quartile, methods of Luo et al. [11] and Wan et al. [12] were applied to estimate the mean and standard deviation of samples [13, 14].

### Quality evaluation

Two researchers independently browsed the title and abstract of the retrieved literature, and read the full text of the literature that may meet the inclusion criteria, and finally determine whether to include them. If there were any disagreements especially on quality assessment, it was resolved by discussion with a third senior clinician. All selected studies were previously published, so there was no need for ethical review and approval or patients consent.

The quality assessment of diagnostic accuracy studies (QUADAS-2) recommended by Cochrane Collaboration was adopted as the evaluation criterion [15]. QUADAS-2 consists of the following key aspects: patient selection, index test, gold standard, flow and timing. Each of them was assessed in terms of risk of bias and signaling questions (yes/no/unclear) were included to assist in judgments. When the criterion is yes, the score increases by 1 point.

### Data analysis

#### Heterogeneity test

Heterogeneity caused by different type of research design, age and gender of patients, pathological subtypes and other variables is a critical factor influencing the accuracy of results. The existence non-threshold effect was tested by Q-test and *I*^*2*^ value using RevMan 5.3 Software. *I*^*2*^<50% indicates insignificant heterogeneity, and a fixed-effect model was applied to merge statistics. *I*^*2*^ ≥ 50% indicates substantial heterogeneity, and a random-effect model was used to merge statistics. Q-test level was *P* < 0.05.

#### Meta-analysis

RevMan 5.3 Software (Cochrane Collaboration, Oxford, UK) was used to calculate the effect size and 95% CI. The pooled sensitivity, specificity, positive likelihood ratio, negative likelihood ratio, diagnostic ratio, AUC and its 95% CI were calculated by Stata 13.1, and SROC curves were constructed.

#### Publication bias

Publication bias was evaluated with Deek’s funnel plot by Stata 13.1 software. *P* > 0.1 indicated that there was no publication bias.

#### Sensitivity analysis

The stability of included studies was evaluated. We eliminated an individual study and calculated the pooled effect of the rest of studies.

## Results

### Literature retrieval results

**Fig. 1:**
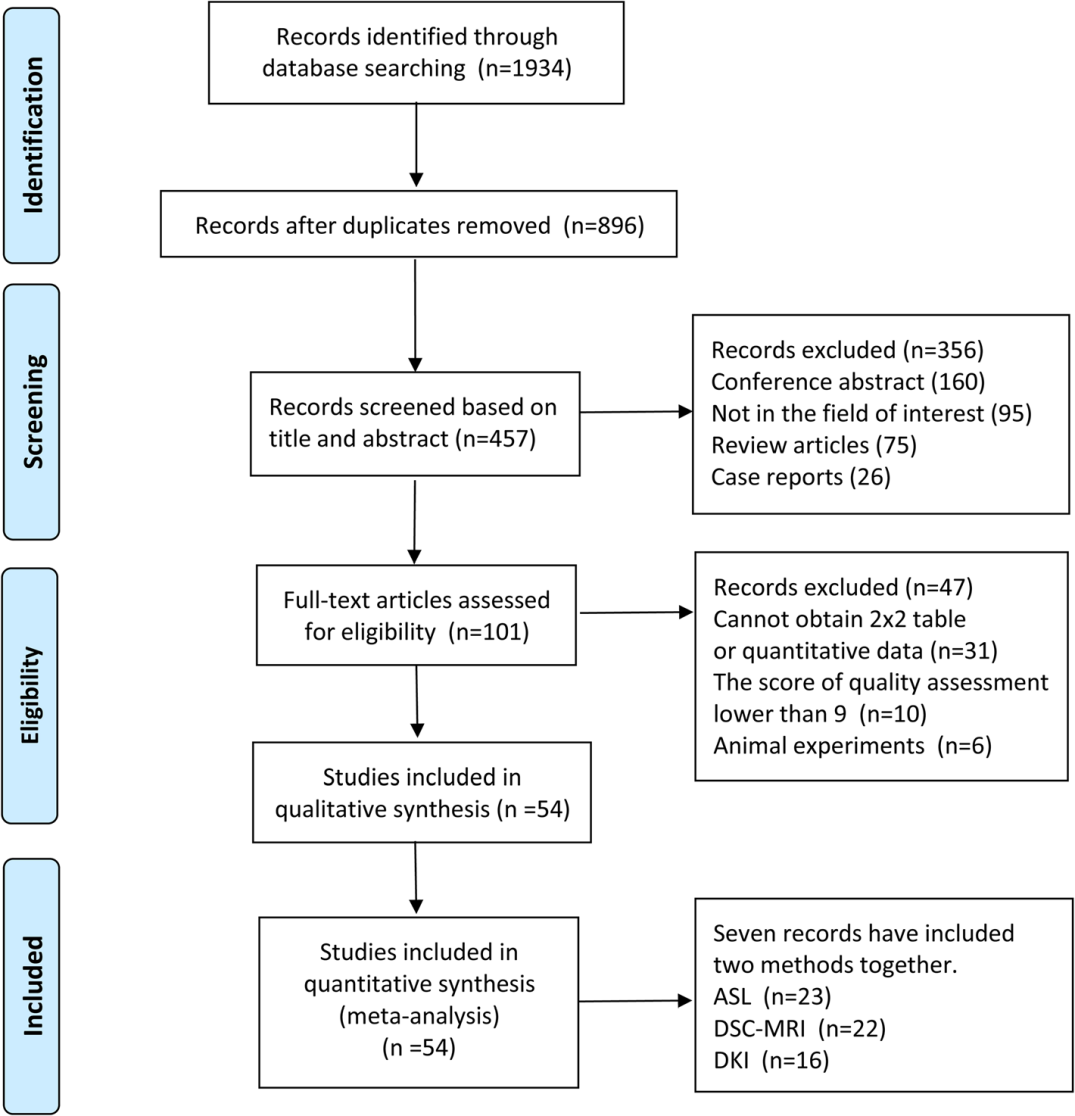
Flow chart of literature screening and identification process

Fifty-four studies were selected for inclusion after reading the full text, of which 43 were in English and 11 were in Chinese. Patients include both adults and children. The studies were conducted in the following countries: China (*n* = 24), India (n = 2), Italy (*n* = 3), Spain (*n* = 1), Turkey (*n* = 1), Sweden (n = 2), Japan (n = 3), Norway (*n* = 1), the United States (*n* = 5), Canada (n = 1), Korea (n = 2), France (n = 1), Germany (*n* = 4). Denmark (n = 1), Belgium (n = 1), Brazil (n = 1), Australia (n = 1). Seven studies reported two methods. Of those studies including quantitative data and Continuous Variable Forest Map, 20 was in ASL, 22 in DSC-MRI, 15 in DKI. Of those studies including fourfold table data for meta-analysis of diagnostic tests, 19 was in ASL, 19 in DSC-MRI, 16 in DKI. The flowchart of retrieval process is presented in Fig. 1. The basic information of the literature included is presented in Table 1.

**Table 1:** Basic information of included studies

First author	Region	Average or median age	n	Gliomas grade(n)	Instrument Type and Field Strength	Technique	Diagnostic parameters	QUADAS-2 score
Arisawa 2018 [16]	Japan	50(19–74)	34	I + II(15),III + IV(19)	GE 3.0 T	ASL,DSC	rCBF, rCBV	12
Cebeci 2014 [17]	Turkey	47 ± 14	33	I + II(13),III + IV(20)	Philips 3.0 T	ASL	rCBF	13
Fudaba 2014 [18]	Japan	59.8 ± 16.8	32	II(9),III(8),IV(15)	Siemens 3.0 T	ASL	rCBF	13
Furtner 2014 [19]	Australia	54 ± 17	33	II(7),III(7),IV(19)	Siemens 3.0 T	ASL	rCBF	12
Jiang J 2014 [20]	China	42.7 ± 15.3	23	I + II(10),III + IV(13)	GE 3.0 T	ASL	rCBF	12
Kim 2008 [21]	South Korea	43(19–74)	61	I + II(26),III + IV(35)	GE 1.5 T	ASL	rCBF	11
Liao H 2016 [22]	China	40.5	41	I + II(20),III + IV(21)	GE 3.0 T	ASL	rCBF	11
Liu 2014 [23]	China	8–75	38	I(5),II(17),III(7),IV(9)	GE 3.0 T	ASL	rCBF	12
Ma 2017 [24]	China	46 ± 18	50	I + II(27),III + IV(23)	GE 3.0 T	ASL,DSC	rCBF, rCBV	11
Qiao F 2015 [25]	China	50(26–72)	28	I + II(11),III + IV(17)	GE 3.0 T	ASL	rCBF	10
Roy 2013 [7]	India	43	64	I(3),II(23),III(9),IV(29)	GE 3.0 T	ASL	rCBF	11
Shen 2016 [26]	China	42	52	I + II(25),III + IV(27)	GE 3.0 T	ASL	rCBF	9
Tian Q 2015 [27]	China	47(19–76)	45	I + II(19),III + IV(26)	GE 3.0 T	ASL	rCBF	12
Wang N 2019 [28]	China	48(23–81)	53	I(1),II(15),III(13),IV(24)	GE 3.0 T	ASL	rCBF	10
Morana 2018 [29]	Italy	9(2–17)	37	I(8),II(14),III(6),IV(9)	Philips 1.5 T	ASL,DSC	rCBF, rCBV	10
Wolf 2005 [30]	USA	50 ± 12	26	I + II(7),III + IV(19)	Siemens 3.0 T	ASL	rCBF	10
Xiao 2015 [31]	China	43.3(6–74)	43	I + II(19),III + IV(24)	GE 3.0 T	ASL	rCBF	11
Yang 2016 [32]	China	51 ± 15.34	43	II(15),III(15),IV(13)	Siemens 3.0 T	ASL	rCBF	12
Zeng 2017 [33]	China	50 ± 13	58	II(13),III(17),IV(28)	GE 3.0 T	ASL	rCBF	11
Zhao J 2016 [34]	China	42(15–64)	18	I + II(8),III + IV(10)	GE 3.0 T	ASL	rCBF	9
Van Cauter 2014 [35]	Belgium	55	31/ 35	I + II(12),III + IV(19)-DSC I + II(13),III + IV(22)-DKI	Philips 3.0 T	DSC ,DKI	rCBV, MK	9
Awasthi 2012 [36]	India	16–65	76	I + II(21),III + IV(55)	GE 1.5 T	DSC	rCBV	12
Boxerman 2016 [37]	USA	52(19–80)	43	II(11),III(9),IV(23)	GE 1.5 T	DSC	rCBV	12
Brendle 2018 [38]	Germany	23–79	41	I + II(24),III + IV(17)	Siemens 3.0 T	DSC	rCBV	14
Catalaa 2006 [39]	USA	23–78	17	II(8),III(9)	GE 1.5 T	DSC	rCBV	11
Cuccarini 2016 [40]	Italy	39.6 ± 12.6	68	I + II(42),III + IV(26)	Siemens 1.5 T	DSC	rCBV	12
Dallery 2017 [41]	France	9.4 (2.1–17.9)	30	I(7),II(4),III(7),IV(12)	GE 3.0 T	DSC	rCBV	11
Falk 2014 [42]	Sweden	22–79	25	II(18),III(7)	Philips 3.0 T	DSC	rCBV	10
Fatima 2014 [43]	Brazil	36.23± 16.95	38	I + II(16),III + IV(22)	GE 1.5 T	DSC	rCBV	13
Hilario 2012 [44]	Spain	23–79	162	II(32),III(29),IV(101)	GE 3.0 T	DSC	rCBV	10
Huang 2015 [45]	China	45(17–72)	35	I(2),II(12),III(9),IV(12)	Siemens 3.0 T	DSC	rCBV	9
Kim 2013 [46]	South Korea	35	63	II(9),III(16),IV(38)	Siemens 3.0 T	DSC	rCBV	9
Law 2006 [47]	USA	42(4–85)	73	II(31),III(16),IV(26)	Siemens 3.0 T	DSC	rCBV	11
Wang M 2011 [48]	China	42.9 ± 14.7	23	I(1),II(5),III(8),IV(9)	Siemens 3.0 T	ASL,DSC	rCBF, rCBV	9
Nguyen 2016 [49]	Canada	54	43	I + II(10),III + IV(33)	Siemens 3.0 T	DSC	rCBV	12
Santarosa 2016 [8]	Italy	55.4 (22–79)	26	II(9),III(4),IV(13)	Philips 3.0 T	DSC	rCBV	9
Server 2011 [50]	Norway	57.73± 12.95	79	II(18),III(14),IV(47)	GE 3.0 T	DSC	rCBV	10
Togao 2017 [51]	Japan	14–75	34	I + II(20),III + IV(14)	Philips 3.0 T	DSC	rCBV	12
Wang X 2016 [52]	China	41 ± 15	37	I + II(14),III + IV(23)	GE 3.0 T	ASL,DKI	rCBF, MK	10
Falk Delgado 2017 [53]	Sweden	48 ± 15	35	II(23),III(12)	Philips 3.0 T	DKI	MK	10
Gao A 2017 [54]	China	48.66± 13.42	34	II(21),III(13)	Siemens 3.0 T	DKI	MK	10
Hempel 2017 [55]	USA	50 ± 14	50	II(25),III(15),IV(10)	Siemens 3.0 T	DKI	MK	11
Jiang 2015 [56]	China	41 ± 14	74	I(3),II(31),III(19),IV(21)	GE 3.0 T	DKI	MK	10
Li 2016 [57]	China	47	37	I + II(16),III + IV(21)	Siemens 3.0 T	DKI	MK	9
Lin 2018 [58]	China	42–75	96	I + II(84),III + IV(12)	GE 3.0 T	DKI	MK	10
Maximov 2017 [59]	Germany	18–59	24	II(8),III(8),IV(8)	GE 3.0 T	DKI	MK	11
Qi 2018 [60]	China	11–67	39	I + II(13),III + IV(26)	Siemens 3.0 T	DKI	MK	9
Raab 2010 [61]	Germany	56	18	II(5),III(13)	Siemens 3.0 T	DKI	MK	11
Raja 2016 [62]	Germany	35	18	II(9),III(9)	Philips 3.0 T	DKI	MK	12
Tan 2016 [63]	China	50	60	I + II(25),III + IV(35)	GE 3.0 T	DKI	MK	11
Tietze 2015 [64]	Denmark	42	34	II(12),III(7),IV(15)	Siemens 3.0 T	DKI	MK	10
Zheng G 2014 [65]	China	40.3 ± 19.5	21	I(2),II(3),III(7),IV(9)	GE 3.0 T	ASL,DSC	rCBF, rCBV	10
Wang Y 2017 [66]	China	47.5 (25–75)	32	I + II(14),III + IV(18)	Siemens 3.0 T	DKI	MK	10
Zhao 2019 [67]	China	25–75	52	II(24),III(8),IV(20)	Siemens 3.0 T	DKI	MK	12

### Analysis

#### rCBF in ASL

**Fig. 2:**
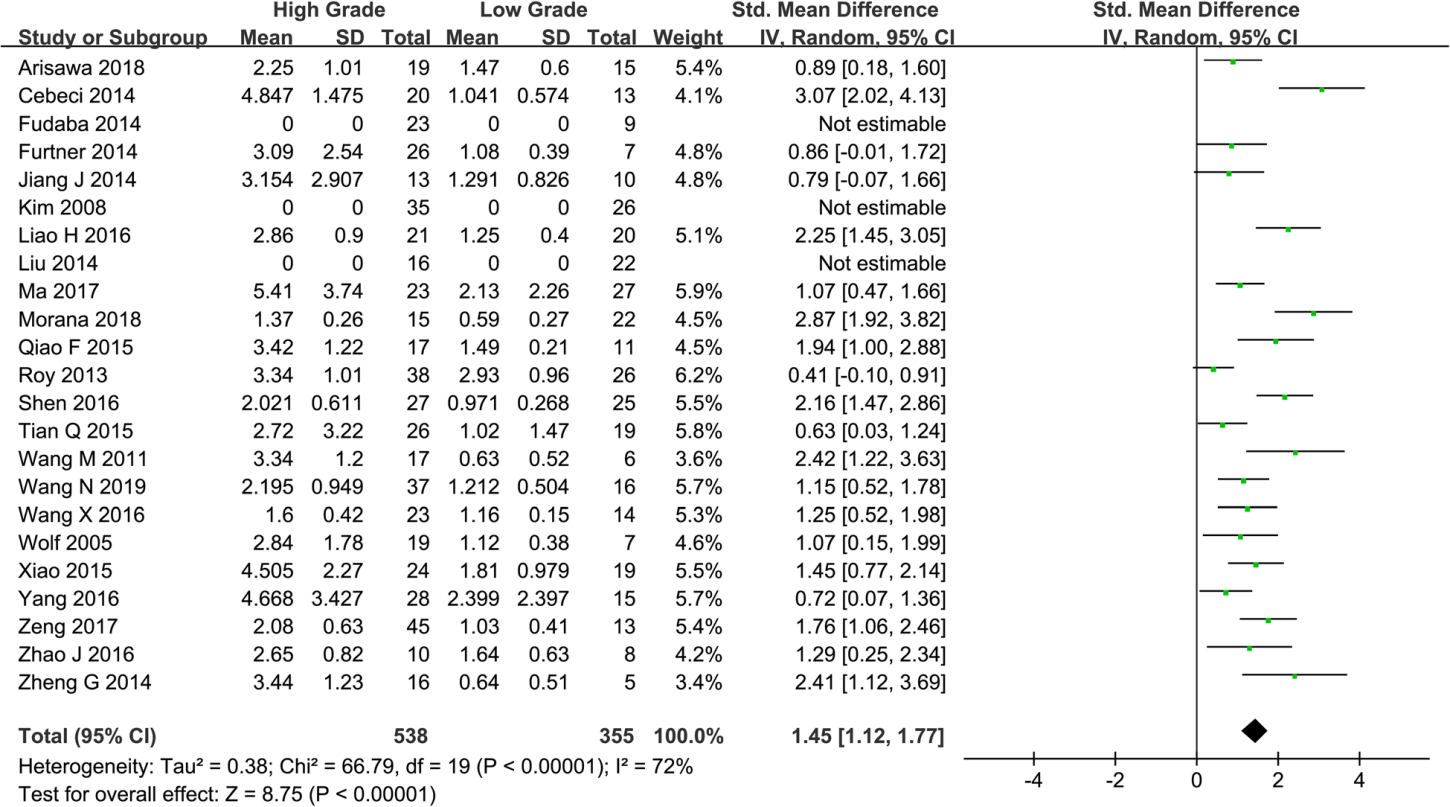
Forest plot of mean difference in rCBF between HGGs and LGGs in ASL. Positive results were observed between HGGs and LGGs

Twenty studies assessing the difference of rCBF between HGGs and LGGs were included. Heterogeneity test showed that *χ*^*2*^ = 66.79, *I*^*2*^ = 72%, *P* < 0.001, indicating substantial heterogeneity. Therefore, the random effect model was applied to estimate the pooled rCBF. The pooled rCBF was 1.45 (1.12, 1.77), *P* < 0.001 (Fig. 2).

#### rCBV in DSC-MRI

**Fig. 3:**
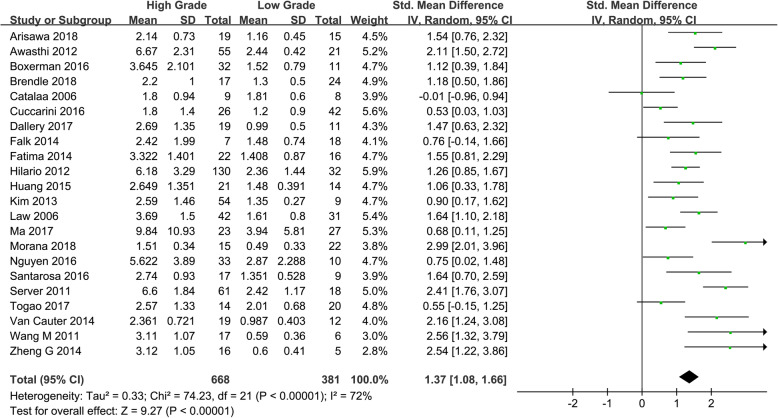
Forest plot of mean difference in rCBV between HGGs and LGGs in DSC-MRI. Positive results were observed between HGGs and LGGs

Twenty-two studies assessing the difference of rCBV between HGGs and LGGs were included. Heterogeneity test showed that *χ*^*2*^ = 74.23, *I*^*2*^ = 72%, *P* < 0.001, indicating substantial heterogeneity. Therefore, the random effect model was applied to estimate the pooled rCBV. The pooled rCBV was 1.37 (1.08, 1.66), *P* < 0.001 (Fig. 3).

#### MK in DKI

**Fig. 4:**
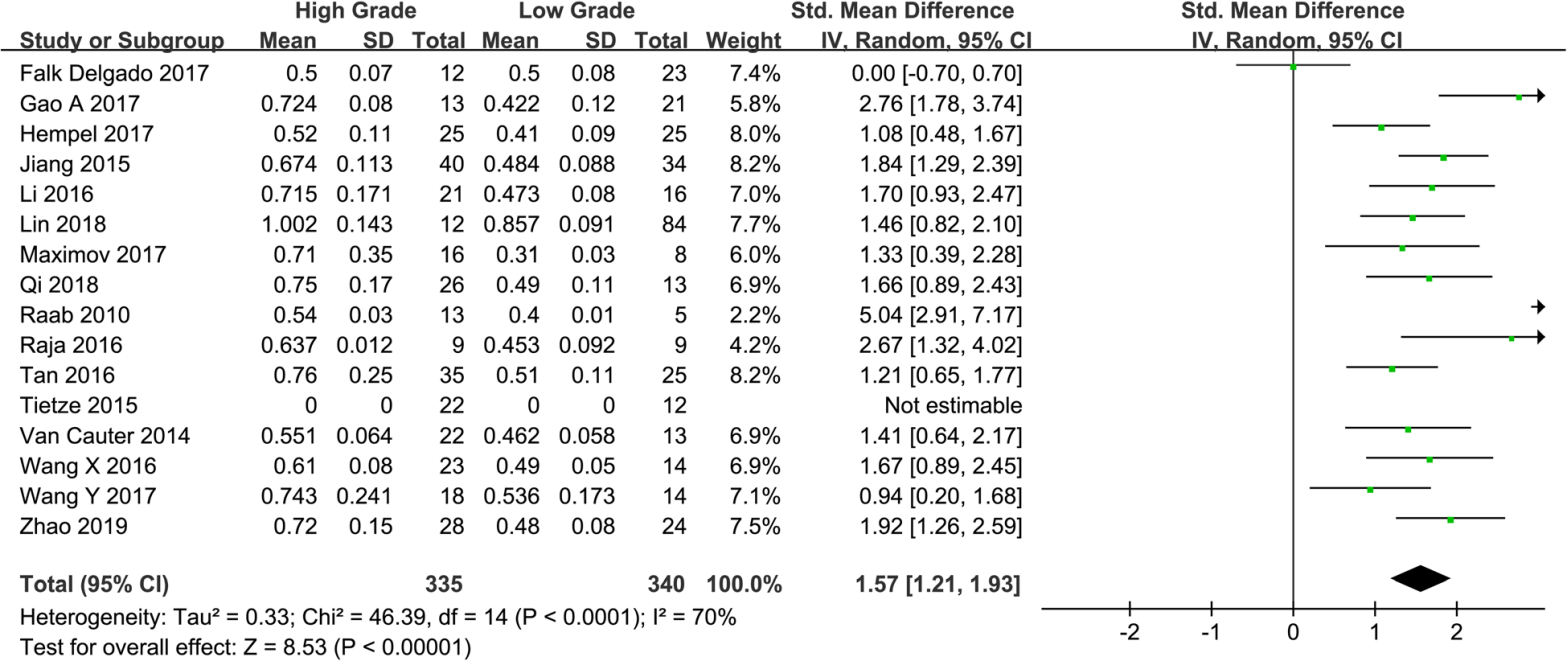
Forest plot of mean difference in MK between HGGs and LGGs in DKI. Positive results were observed between HGGs and LGGs

Fifteen studies assessing the difference of MK between HGGs and LGGs were included. Heterogeneity test showed that *χ*^*2*^ = 46.39, *I*^*2*^ = 70%, *P* < 0.001, indicating substantial heterogeneity. Therefore, the random effect model was applied to estimate the pooled MK. The pooled MK was 1.57 (1.21, 1.93), *P* < 0.001 (Fig. 4).

#### Diagnostic value

**Fig. 5:**
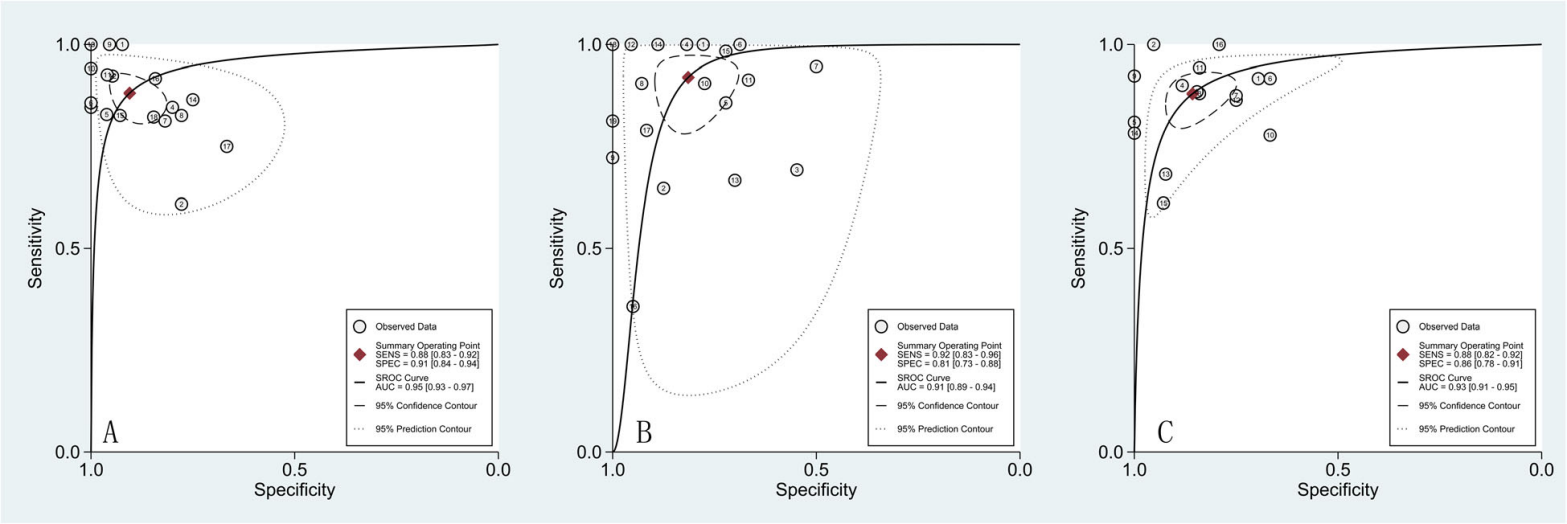
SROC Curve for Each Parameter in the Grading of Cerebral Gliomas. A.ASL, B. DSC-MRI, C.DKI

Sensitivity, specificity, positive likelihood ratio, negative likelihood ratio, diagnostic ratio and area under curve were summarized according to the studies including fourfold table (Table 2). The results showed that rCBF had the highest diagnostic ratio (DOR) of 71 (31,163). The SROC curve suggested that rCBF had the highest AUC value of 0.95 (0.93,0.97), followed by MK 0.93 (0.91,0.95), and rCBV 0.91 (0.89,0.94) (Fig. 5).

**Table 2:** The values of rCBF, rCBV and MK

index	n	Sen (95% CI)	Spe (95% CI)	PLR (95% CI)	NLR (95% CI)	DOR (95% CI)	AUC (95% CI)
rCBF	19	0.88 (0.83,0.92)	0.91 (0.84,0.94)	9.3 (5.4,16.0)	0.13 (0.09,0.20)	71 (31,163)	0.95 (0.93,0.97)
rCBV	19	0.92 (0.83,0.96)	0.81 (0.73,0.88)	5.0 (3.3,7.4)	0.10 (0.05,0.22)	50 (20,129)	0.91 (0.89,0.94)
MK	16	0.88 (0.82,0.92)	0.86 (0.78,0.91)	6.2 (4.1,9.3)	0.14 (0.10,0.21)	44 (26,75)	0.93 (0.91,0.95)

**Fig. 6:**
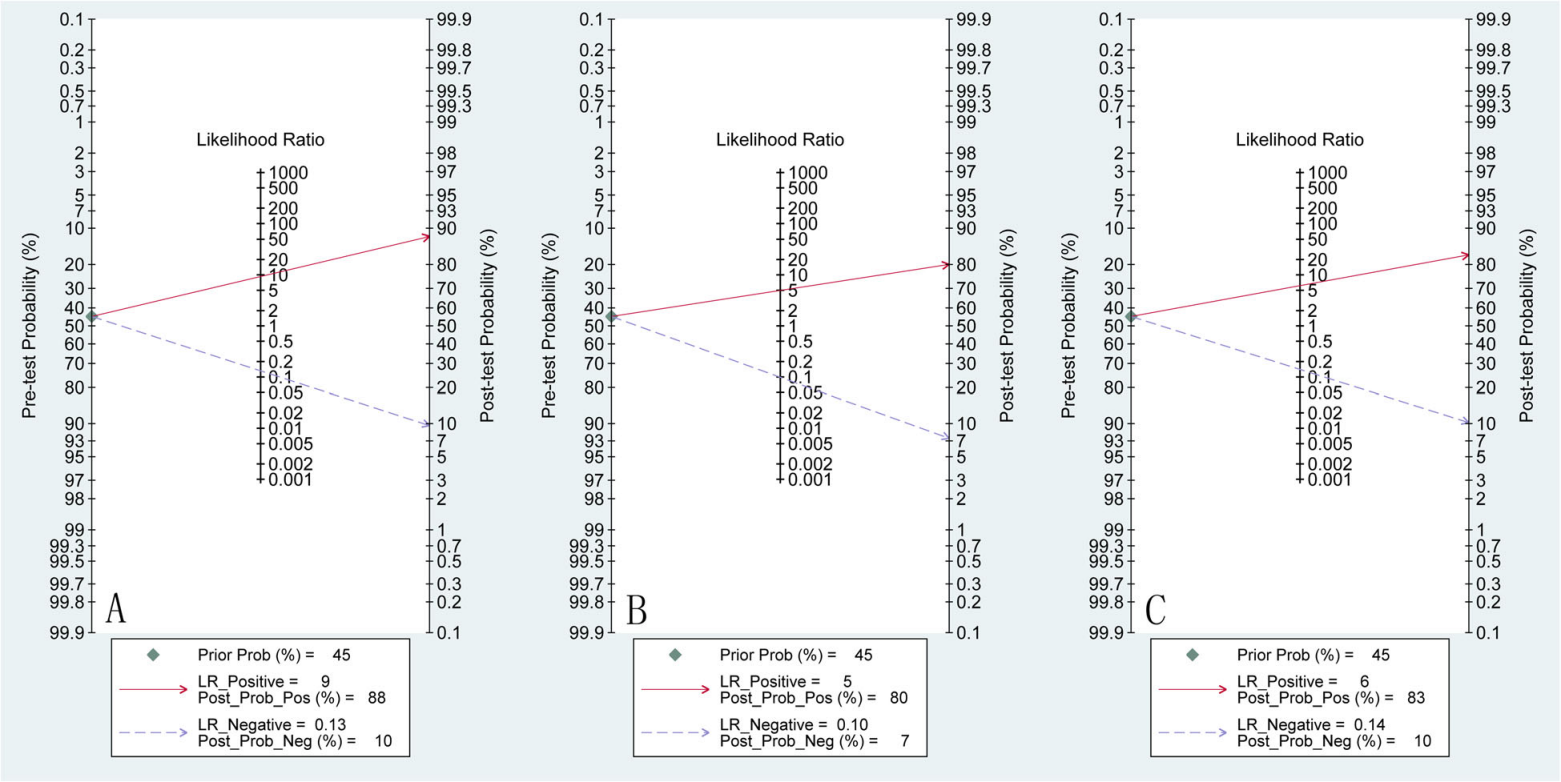
Fagan Map for Each Parameter in the Grading of Cerebral Gliomas. A.ASL, B. DSC-MRI, C.DKI

The incidence of gliomas is about 45% of all intracranial tumors [63]. The Fagan diagram of rCBF, rCBV and MK in the grading of gliomas is shown in Fig. 6. Compared with 45% pre-test probability, the post-test probability of rCBF, rCBV and MK increases to 88, 80 and 83%, respectively. The DOR value of rCBF is 71 (31,163), indicating a high pooled diagnostic accuracy.

#### Meta-regression

The results of meta-regression are shown in Table 3. Among the five covariates in ASL study, region, year of study, number of patients and QUADAS-2 score were all important factors contributing to heterogeneity except for field strength. Among the six covariates in DSC-MRI study, region, year of study, number of patients, field strength and QUADAS-2 score, none had significant impact on heterogeneity. Among the five covariates in DKI study, the year of study, age of patients, number of patients and QUADAS-2 score all had no significant impact on heterogeneity except for region.

**Table 3:** Meta-regression

	Variable	Subgroup	n	Overall estimate of meta-regression			
				Sensitivity(95% CI)	p	Specificity(95% CI)	p
ASL	Region	China	14	0.89(0.84,0.94)	0.01	0.89(0.83,0.95)	0.71
		others	5	0.86(0.77,0.95)		0.94(0.88,1.00)	
	Year	2008–2014	8	0.87(0.80,0.93)	0.00	0.88(0.80,0.96)	0.01
		2015–2019	11	0.89(0.83,0.95)		0.92(0.87,0.98)	
	Number of patients	≤40	10	0.90(0.84,0.95)	0.00	0.93(0.87,0.99)	0.01
		>40	9	0.87(0.81,0.93)		0.88(0.81,0.95)	
	Field strength	1.5 T	2	0.90(0.79,1.00)	0.21	0.96(0.90,1.00)	0.13
		3.0 T	17	0.88(0.83,0.93)		0.89(0.84,0.94)	
	QUADAS-2 score	≤10	7	0.93(0.89,0.98)	0.00	0.94(0.89,1.00)	0.01
		>10	12	0.84(0.79,0.90)		0.88(0.81,0.94)	
DSC-MRI	Region	China	4	0.94(0.83,1.00)	0.77	0.88(0.74,1.00)	0.14
		others	15	0.91(0.84,0.99)		0.80(0.72,0.88)	
	Year	2006–2014	10	0.95(0.89,1.00)	0.07	0.80(0.69,0.90)	0.20
		2015–2017	9	0.87(0.74,0.99)		0.83(0.73,0.93)	
	Age	≤45	9	0.94(0.87,1.00)	0.31	0.85(0.75,0.95)	0.03
		>45	10	0.90(0.80,1.00)		0.79(0.68,0.89)	
	Number of patients	≤40	10	0.95(0.88,1.00)	0.33	0.89(0.82,0.95)	0.56
		>40	9	0.89(0.79,1.00)		0.72(0.62,0.83)	
	Field strength	1.5 T	4	0.98(0.93,1.00)	0.23	0.76(0.60,0.92)	0.00
		3.0 T	15	0.90(0.82,0.98)		0.83(0.75,0.91)	
	QUADAS-2 score	≤10	10	0.94(0.88,1.00)	0.14	0.86(0.77,0.95)	0.01
		>10	9	0.88(0.77,0.99)		0.77(0.66,0.88)	
DKI	Region	China	9	0.89(0.83,0.95)	0.01	0.89(0.82,0.95)	0.01
		others	7	0.86(0.77,0.94)		0.81(0.69,0.92)	
	Year	2010–2015	4	0.85(0.75,0.95)	0.14	0.89(0.79,0.99)	0.06
		2016–2019	12	0.89(0.84,0.94)		0.85(0.77,0.96)	
	Age	≤48	9	0.85(0.78,0.92)	0.14	0.87(0.79,0.94)	0.02
		>48	7	0.91(0.85,0.97)		0.84(0.75,0.93)	
	Number of patients	≤40	11	0.83(0.77,0.89)	0.32	0.88(0.81,0.94)	0.00
		>40	5	0.93(0.89,0.98)		0.80(0.70,0.90)	
	QUADAS-2 score	≤10	10	0.84(0.78,0.91)	0.18	0.87(0.80,0.94)	0.02
		>10	6	0.92(0.87,0.97)		0.83(0.72,0.93)	

#### Subgroup analysis

Subgroup analysis was successively carried out according to the region and technique in ASL, the region and magnetic resonance field strength in DSC-MRI, and the region in DKI. The results of subgroup analysis are shown in Table 4.

**Table 4:** Subgroup analysis

	Subgroup	Category	n	p	Sen (95% CI)	Spe (95% CI)	PLR (95% CI)	NLR (95% CI)	DOR (95% CI)	AUC (95% CI)
ASL	Region	CHINA	14	0.30	0.88 (0.83,0.92)	0.89 (0.81,0.94)	8.4 (4.5,15.4)	0.13 (0.09,0.20)	63 (25,157)	0.94 (0.92,0.96)
		other	5	0.35	0.89 (0.68,0.97)	0.94 (0.84,0.98)	14.0 (5.1,38.2)	0.11 (0.03,0.40)	123 (18,846)	0.96 (0.94,0.98)
	Technique	3D PCASL	10	0.33	0.87 (0.82,0.91)	0.88 (0.81,0.93)	7.6 (4.4,13.1)	0.14 (0.10,0.21)	53 (24,116)	0.92 (0.90,0.94)
		PASL	8	0.46	0.93 (0.75,0.98)	0.93 (0.80,0.98)	14.2 (4.2,48.1)	0.08 (0.02,0.31)	183 (19,1754)	0.98 (0.96,0.99)
DSC-MRI	Region	CHINA	4	0.23	0.91 (0.82,0.96)	0.90 (0.55,0.99)	9.6 (1.5,60.4)	0.10 (0.05,0.21)	95 (11,835)	0.89 (0.82,0.96)
		other	15	0.00	0.92 (0.80,0.97)	0.80 (0.70,0.87)	4.6 (3.1,6.9)	0.10 (0.04,0.26)	46 (15,144)	0.90 (0.87,0.92)
	Field strength	3.0 T	15	0.00	0.88 (0.79,0.94)	0.82 (0.73,0.89)	5.0 (3.2,7.7)	0.14 (0.08,0.26)	35 (17,73)	0.91 (0.88,0.93)
		1.5 T	4	0.01	1.00 (0.12,1.00)	0.77 (0.57,0.90)	4.4 (2.1,9.1)	0.00 (0.00,10.67)	2976 (0,3125)	0.91 (0.89,0.93)
DKI	Region	CHINA	9	0.00	0.90 (0.81,0.95)	0.90 (0.79,0.95)	8.6 (4.3,17.2)	0.11 (0.06,0.21)	75 (35,160)	0.95 (0.93,0.97)
		other	7	0.46	0.85 (0.76,0.91)	0.79 (0.69,0.87)	4.1 (2.7,6.2)	0.20 (0.12,0.32)	21 (10,44)	0.88 (0.85,0.91)

### Publication bias

**Fig. 7:**
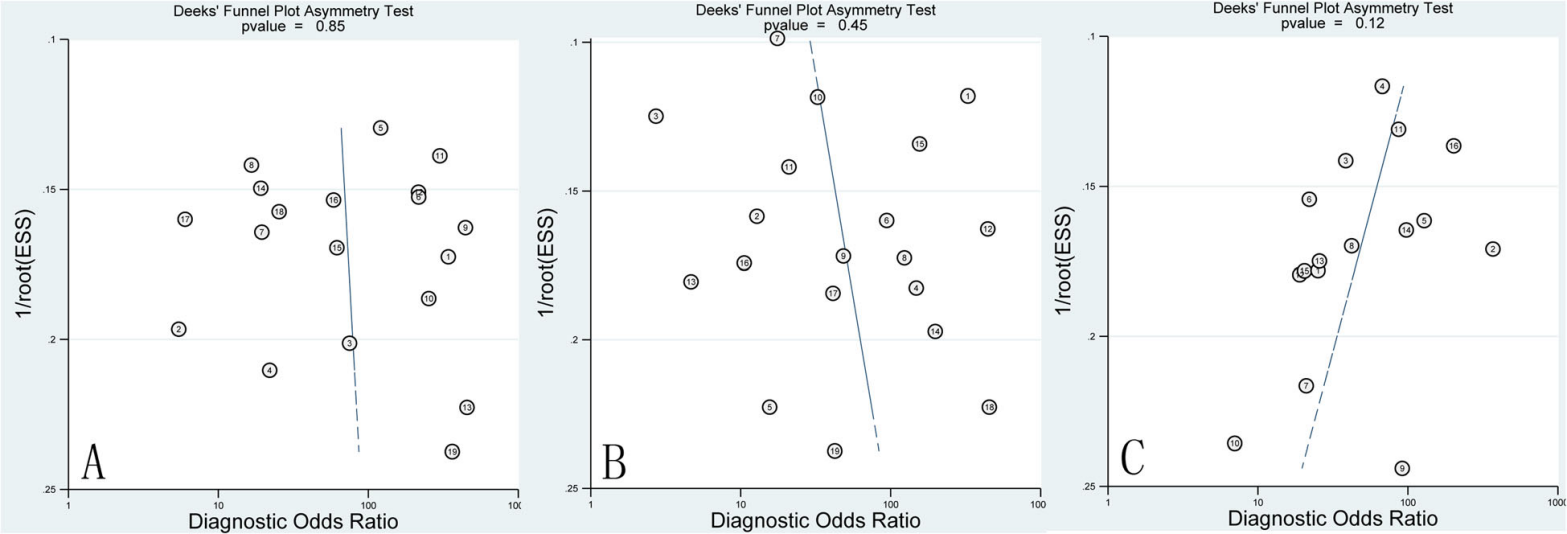
Funnel plot of publication bias. **a** ASL group; (**b**) DSC-MRI group; (**c**) DKI group

Deek’s test was used to evaluate publication bias for studies containing fourfold Tables. *P* > 0.1 indicated that there was no publication bias. 19 studies of ASL, 19 studies of DSC-MRI, 16 studies of DKI were eligible for Deek’s test. Deeks funnel plot (Fig. 7) showed no significant publication bias for all groups (*P* = 0.85, *P* = 0.45, *P* = 0.12, for ASL, DSC-MRI, DKI group, respectively).

### Sensitivity analysis

Sensitivity analysis is a key method for assessing heterogeneity and publication bias. We eliminated an individual study and calculated the pooled effect of the rest of studies. Compared with the pooled effect of all the included studies, we could determine the influence of individual study on the pooled effect. Results of this meta-analysis revealed that the included studies had no significant changes on the pooled value of rCBF and rCBV. However, the MK of Delgado et al. [53] showed significant influence on heterogeneity and publication bias before it was eliminated (*I*^*2*^ = 70% to *I*^*2*^ = 54% calculated by Revman5.3).

## Discussion

This meta-analysis revealed the pooled rCBF, rCBV and MK of HGGs were higher than those of LGGs, with the results statistically significant. The specificity of rCBF is the highest among all parameters, suggesting that the rate of misdiagnosis in rCBF is the lowest. The sensitivity of rCBV is the highest, suggesting that the rate of missed diagnosis in rCBV is the lowest. The results of meta-regression showed that there were many factors contributing to the heterogeneity of ASL studies, while the studies of DSC-MRI and DKI were relatively stable. Although three kinds of MRI techniques included in this study could be applied to grade gliomas, the DOR suggested that rCBF in ASL had the highest diagnostics accuracy.

DSC-MRI perfusion imaging uses an exogenous contrast agent and relies on the acquisition of T2* images. DSC-MRI detects changes in MR signal as the contrast agent passes through the blood vessels, thus haemodynamic parameter (rCBV) can be indicative of microvascular properties such as vascular flow [8]. Compared to LGGs, HGGs have more abundant blood supply; therefore, the hemodynamic parameters (rCBV) would manifest notable increases significantly, which is consistent with the findings of Winkler et al. [68]. Awasthi et al. [36] observed that the microvessel density (MVD) and the positive expression of vascular endothelial growth factor (VEGF) had significant correlation with the pathological grade of gliomas and the rCBV value. Although the range of rCBV values reported in the literature amongst various types of gliomas, the most researchers observed higher rCBV in HGGs [69]. In this meta-analysis, we found that the discriminative values of sensitivity and specificity were 92 and 81% by rCBV between HGGs and LGGs.

ASL is a completely non-invasive MRI technique which measures blood flow by using magnetically labeled water protons in arterial blood as an endogenous tracer. It is not affected by the integrity of blood-brain barrier, therefore accurately evaluates gliomas microcirculation information, reflecting the situation of tumor angiogenesis, and thus the gliomas grade can be more accurately assessed [70, 71]. The relative rCBF has been widely used to discriminate between LGGs and HGGs. Although ASL suffers from low signal-to-noise ratio as well as sensitivity to motion, Cebeci and Luh et al. reported a strong correlation between ASL-derived CBF values and DSC-derived CBF values in brain tumours [17, 72]. Several studies had revealed that rCBF of ASL was a rigorous parameter of grading gliomas, thereby allowing it an alternative method of DSC-MRI [73–75].

Diffusion kurtosis imaging (DKI), first proposed by Professor Jensen of New York University in 2005, is a technique intending to explore the properties of non-gaussian diffusion of water molecules [76, 77]. It has been proposed to more accurately characterise the complicated water diffusion in biological tissues. The most commonly used parameter of DKI is mean kurtosis (MK) which provides additional information about tumour heterogeneity. The cellular pleomorphism and nuclear polymorphism in HGGs are more significant than those in LGGs. The proliferation of interstitial vessels is also more abundant in HGGs and thus the MK value is higher [9]. Some studies indicated that MK was higher in HGGs. Raab et al. [61] found that the AUC of MK was 92.3% for differentiating HGGs from LGGs, which were in strong agreement with the findings in this meta-analysis.

Heterogeneity is common in meta-analysis. After excluding the research of Falk Delgado et al., the heterogeneity of MK decreased from 70 to 54%. Since there is moderate heterogeneity in this meta-analysis, clinical decisions should be made cautiously based on these results. Heterogeneity may be caused by the following aspects: (1) imbalance in the distribution of HGGs and LGGs: for instance, grade I gliomas were not studied in some research which resulted bias in case selection; (2) different experimental conditions set by researchers, such as different instrument models, parameter settings, post-processing methods, etc. (3) regional heterogeneity resulted from inclusion of literature from different countries and regions; (4) The region of interest (ROI) and the reference region were heterogeneously placed in the different studies, which may have an impact on the results.

The main limitations of this study are: 1. This study only focused on diagnostic value of ASL, DSC-MRI and DKI in distinguishing LGGs from HGGs, their role in the follow-up and each specific pathological grade of gliomas were not discussed; 2. Only research in Chinese and English were included, the sample size was relatively small; 3. Most studies used the WHO classification system without molecule genomics.

## Conclusion

Quantitative parameters rCBF in ASL, rCBV in DSC-MRI and MK in DKI had excellent diagnostic performances for differentiating HGGs from LGGs. rCBF is a rigorous parameter of grading gliomas with AUC of 0.95, thereby allowing it an alternative method of DSC-MRI or DKI.

## Data Availability

Not applicable.

## Abbreviations

ASL: arterial spin labeling
DSC-MRI: dynamic magnetic sensitivity contrast-enhanced magnetic resonance imaging
DKI: diffusion kurtosis imaging
rCBF: cerebral blood flow
rCBV: relative cerebral blood volume
MK: mean kurtosis
CBM: China Biology Medicine disc
CNKI: China National Knowledge Infrastructure
3DpCASL: three-dimensional pseudo-continuous arterial spin labeling
CT: computed tomography
PET: positron emission tomography
